# Factors Associated with In-Hospital Mortality after Continuous Renal Replacement Therapy for Critically Ill Patients: A Systematic Review and Meta-Analysis

**DOI:** 10.3390/ijerph17238781

**Published:** 2020-11-26

**Authors:** Hyeon-Ju Lee, Youn-Jung Son

**Affiliations:** 1 Department of Nursing, Tongmyong University, Busan 48520, Korea; lhj209@tu.ac.kr; 2 Red Cross College of Nursing, Chung-Ang University, Seoul 06974, Korea

**Keywords:** continuous renal replacement therapy, critical illness, hospital mortality, risk factor, systematic review

## Abstract

Continuous renal replacement therapy (CRRT) is a broadly-accepted treatment for critically ill patients with acute kidney injury to optimize fluid and electrolyte management. Despite intensive dialysis care, there is a high mortality rate among these patients. There is uncertainty regarding the factors associated with in-hospital mortality among patients requiring CRRT. This review evaluates how various risk factors influence the in-hospital mortality of critically ill patients who require CRRT. Five databases were surveyed to gather relevant publications up to 30 June 2020. We identified 752 works, of which we retrieved 38 in full text. Finally, six cohort studies that evaluated 1190 patients were eligible. The in-hospital mortality rate in these studies ranged from 38.6 to 62.4%. Our meta-analysis results showed that older age, lower body mass index, higher APACHE II and SOFA scores, lower systolic and diastolic blood pressure, decreased serum creatinine level, and increased serum sodium level were significantly associated with increased in-hospital mortality in critically ill patients who received CRRT. These results suggest that there are multiple modifiable factors that influence the risk of in-hospital mortality in critically ill patients undergoing CRRT. Further, healthcare professionals should take more care when CRRT is performed on older adults.

## 1. Introduction

Acute kidney injury (AKI) is characterized by elevated plasma creatinine level and decreased urine output and is often accompanied by multiple comorbidities, such as old age, congestive heart failure, diabetes, hypertension, and stroke [1,2]. AKI is associated with significantly high hospital mortality and morbidity among critically ill patients [2,3]. The prevalence of AKI has been reported for approximately 30–60% critically ill patients in intensive care units (ICUs) [4,5]. Therefore, renal replacement therapy is vital for critically ill patients with AKI to provide supportive management in critical care settings aimed at speeding up renal recovery and preventing adverse events [6]. The first choice for patients with AKI is continuous renal replacement therapy (CRRT), as most critically ill patients are hemodynamically un-stable [7,8]. CRRT refers to either dialysis or filtration treatments that operate continuously [2]. CRRT is a predominant form of renal replacement therapy and has proven to be an effective treatment of ICU patients with AKI with multi-organ failure [1,9]. Numerous patients in critical care settings experience AKI for various reasons and consequently require renal dialysis; many of these patients receive CRRT, due to its advantages regarding hemodynamic stability, accurate volume control, and steady acid-base and electrolyte correction [7]. Furthermore, providing CRRT is standard practice for critically ill patients with multi-organ failure—including AKI—in most ICUs worldwide [1,2].

Despite CRRT’s significant advantage over intermittent renal replacement therapy, it has certain drawbacks. It is usually implemented over 24 h to several days and is an inherently complex process, with the requirement for anticoagulation and the use of high volumes of fluid [10,11,12]. In addition, patients are kept immobile in bed with multiple intravenous access tubes, ventilation equipment, and other support equipment, while receiving CRRT; these circumstances are associated with prolonged hospitalization, increased financial burden, and increased mortality rate [13,14]. Patients in ICU who are receiving CRRT are often presumed to have poor prospective outcomes, which results in their care not being escalated to the appropriate level [12]. Recent studies have reported that the mortality associated with AKI in ICU patients receiving CRRT is too high, at around 64% [15]. Thus, patients with AKI who require CRRT may have the worst short- or long-term prognosis among critically ill patients, and an evidence-based approach is needed to investigate this group. Existing studies focused primarily on assessing in-hospital mortality in patients with AKI who require CRRT [3,4] or on evaluating the strategy of early initiation of CRRT [2,9]. However, current knowledge on which patients are more likely to become vulnerable, due to CRRT, is limited. Specifically, the factors predicting in-hospital mortality in critically ill patients who require CRRT have not been established. Critical care teams should be aware that certain risk factors should be considered in the triage of ICU patients receiving CRRT. Therefore, this systematic review and meta-analysis aimed to integrate existing evidence relating to the pre-dictors of in-hospital mortality of critically ill patients in ICU receiving CRRT.

## 2. Methods

### 2.1. Search Strategies

We performed a systematic review, prospectively registered on PROSPERO (ID: CRD42020211172). This systematic review was conducted following the Preferred Reporting Items for Systematic Reviews and Meta-Analysis (PRISMA) guidelines [16]. The research question was organized in “PICO” format (P = patient, I = interest or intervention, C = comparison, and O = outcomes), and the following question was asked: “What were the associated risk factors (Intervention) for in-hospital mortality (Outcome) of critically ill patients after receiving CRRT treatment (Patient)?” We did not utilize a comparison because this review did not include clinical trials. Literature searches for relevant publications up to 30 June 2020 were conducted using the online databases of PubMed, CINAHL, Web of Science, Embase, and the Cochrane Library. The PICO format was utilized to formulate the research question [17]. Our search strategy was based on Medical Subject Heading (MeSH) and non-MeSH keywords: (“critical illness” or “critical care” or “intensive care unit” or “intensive care” or “ICU”) and (“continuous renal replacement therapy” or “CRRT” or “renal replacement therapy”) and (“mortality” or “death”). We reviewed the list of reference studies already identified to confirm additional studies (Table S1).

### 2.2. Study Selection

We identified 752 works, of which we retrieved 38 in full text. Finally, six cohort studies that evaluated 1190 patients were eligible. Two reviewers (Y.J.S. and H.J.L.) independently assessed the literature retrieval, confirmed potential citations, and extracted information from the included studies. Any disagreements encountered were resolved by discussion. The current review included studies meeting the following conditions: (1) Full-length reports published in English in peer-reviewed journals, (2) study designs that were cohort studies with critically ill patients receiving CRRT (≥18 years), (3) studies with an outcome variable of in-hospital mortality, which was defined as in-hospital death occurring during the initial hospitalization or during rehospitalization, and (4) studies in which an odds ratio (OR) or standard mean difference with a 95% confidence interval (CI) was reported or could be calculated. Excluded studies were: (1) Intervention studies, case reports, editorials, reviews, abstracts, and letters, (2) studies using a hemodialysis modality other than CRRT (e.g., intermittent hemodialysis or sustained low efficient dialysis).

### 2.3. Data Extraction and Synthesis

Two reviewers (Y.J.S. and H.J.L.) extracted the following information from the included studies: Last name of the first author, publication year, study location, study design, follow-up period, participant characteristics (e.g., sample size, mean age, and gender), indication for initiation of CRRT, CRRT modality, in-hospital mortality rate, and risk factors of in-hospital mortality.

### 2.4. Data Analysis

We utilized comprehensive meta-analysis software (version 3.0; Biostat, Englewood, NJ, USA) to calculate random-effects pooled estimates for the association between the 28 meta-analyzable variables and in-hospital mortality. Considering data regarding risk factors, dichotomous variables were represented as ORs with 95% Cis and continuous variables were represented as standardized mean differences (SMD) that were statistically significant. The inverse variance index (I^2^) with its 95% confidence intervals was used to quantitatively assess study heterogeneity. A value of 0–25% indicates no observed heterogeneity, 25–50% low heterogeneity, 50–75% moderate heterogeneity, and >75% high heterogeneity [18].

### 2.5. Assessment of Methodological Quality

For cohort studies, the risk of bias was critically appraised according to the Newcastle-Ottawa Scale (NOS; Table 1) [19]. The NOS employs a star system to rate the selection of studies (0–4 stars); comparability of studies (0–2 stars), and the ascertainment of the outcomes of interest (0–3 stars). Studies were considered to be of high quality with a low-risk bias if the NOS level was ≥6 out of 9 and to be of low quality with a high-risk of bias if the score was ≤3 out of 9. Studies included were independently evaluated by two researchers to assess the methodological quality. Any disagreements between the researchers during the quality assessment were resolved by discussion until consensus was reached.

## 3. Results

### 3.1. Literature Search

**Figure 1:**
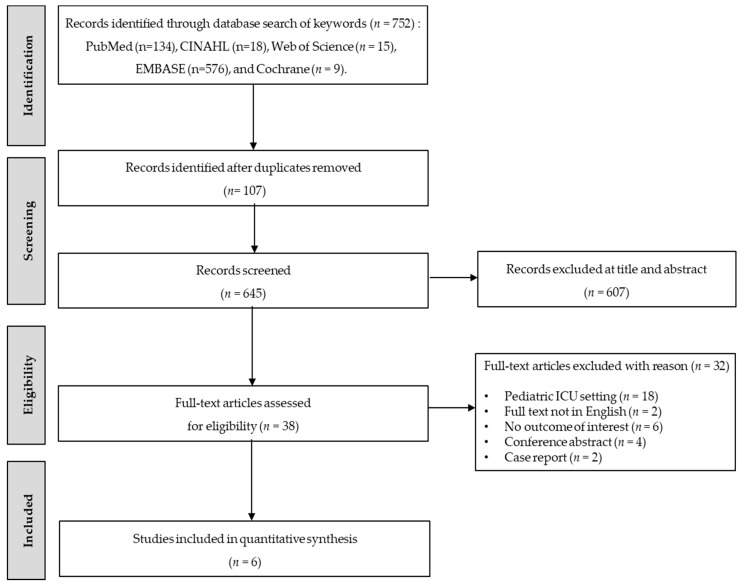
Flow diagram for study selection.

The literature selection process is shown in Figure 1. We identified 752 works (PubMed: 134; CINAHL: 18; Web of Science: 15; EMBASE: 576; and Cochrane: 9). We utilized EndNote (version X7, Thomson Reuters, New York, NY, USA) to remove 107 duplicate citations. After conducting a title and abstract review, 607 articles were excluded, leaving 38 articles to undergo full-text review. Finally, six cohort studies were included for systematic review, and five studies were included in the meta-analysis.

### 3.2. Characteristics of the Included Studies

The characteristics of the included studies are presented in Table 1. Two of the six included studies were designed as prospective studies [20,21], and the other four were retrospective studies [22,23,24,25]. This systematic review comprises 1190 patients from the six studies, 744 males and 446 females. One study did not report the number of participants [22]. The mean ages of the study participants ranged from 57 to 69 years and the follow-up periods ranged from 13 to 109 months. The CRRT modality was categorized as continuous venovenous hemofiltration (CVVH) and continuous venovenous hemodiafiltration (CVVHDF). The in-hospital mortality rate of the study participants ranged from 38.6% to 62.4%. The NOS methodological quality score was between 7 and 9.

### 3.3. Risk Factors for In-Hospital Mortality

We identified 28 risk factors for in-hospital mortality among critically ill patients who received CRRT (Table 2). Eight factors—including age, body mass index (BMI), Acute Physiologic Assessment and Chronic Health Evaluation (APACHE II) score, sequential organ failure assessment (SOFA) score, systolic blood pressure (BP), diastolic BP, serum creatinine level, and serum sodium level—were significantly associated with in-hospital mortality among critically ill patients who received CRRT.

#### 3.3.1. Age

Four studies, including 992 patients, investigated the relationship between increased age and in-hospital mortality among critically ill patients who received CRRT [20,21,23,24]. The pooled results suggested that advanced age was associated with the in-hospital mortality (SMD: 0.26 years; 95% CI: 0.07 to 0.44). The moderate heterogeneity among studies was significant (I^2^ = 47.3%, *p* = 0.127; Table 3).

#### 3.3.2. Body Mass Index

Three studies, including 652 patients, assessed the relationship between BMI and the in-hospital mortality among critically ill patients who received CRRT [20,21,24]. The pooled estimate showed that lower BMI was related to in-hospital mortality (SMD: −0.17 kg/m^2^; 95% CI −0.33 to −0.01). There was no significant heterogeneity among the studies (I^2^ = 3.7%, *p* = 0.354; Table 3).

#### 3.3.3. APACHE II

Three studies involving 752 patients all together investigated the relationship between APACHE II and in-hospital mortality among critically ill patients who received CRRT [20,21,23]. The pooled estimate demonstrated that a higher APACHE II score was associated with in-hospital mortality (SMD: 1.05; 95% CI: 0.36 to 1.75). There was significantly high heterogeneity among the studies (I^2^ = 94.0%, *p* < 0.001; Table 3).

#### 3.3.4. SOFA

Considering the results of three relevant studies that include 752 patients [20,21,23], the pooled estimate showed that a higher SOFA score was associated with in-hospital mortality among critically ill patients who received CRRT (SMD: 1.06; 95% CI: 0.61 to 1.51). The high heterogeneity among the studies was significant (I^2^ = 85.3%, *p* = 0.001; Table 3).

#### 3.3.5. Systolic Blood Pressure

The relationship between systolic BP and in-hospital mortality among critically ill patients who received CRRT was evaluated in two studies involving 752 patients collectively [23,24]. The results suggested that decreased systolic BP was associated with in-hospital mortality (SMD: 0.38 mmHg; 95% CI: −0.55 to −0.22). There was no significant heterogeneity in either of these studies (I^2^ = 1.2%, *p* = 0.314; Table 3).

#### 3.3.6. Diastolic Blood Pressure

Two studies collectively, including 725 patients, investigated the relationship between diastolic BP and in-hospital mortality among critically ill patients who received CRRT [23,24]. The overall results showed that decreased diastolic BP was associated with in-hospital mortality (SMD: 0.27 mmHg; 95% CI: −0.43 to −0.10). There was no significant heterogeneity in either of these studies (I^2^ = 0%, *p* = 0.555; Table 3).

#### 3.3.7. Serum Creatinine Level

An analysis of the serum creatinine level was performed by three studies involving 752 patients [20,21,23]. The pooled estimate suggested that a decreased serum creatinine level was associated with in-hospital mortality among patients who received CRRT (SMD: −0.34 mg/dL; 95% CI: −0.48 to −0.19). There was no significant heterogeneity among these studies (I^2^ = 0%, *p* = 0.541; Table 3).

#### 3.3.8. Serum Sodium Level

Two studies involving 580 patients performed analyses of serum sodium level and were used in this review [23,24]. The pooled estimate suggested that increased serum sodium level was associated with in-hospital mortality (SMD: 0.21 mmol/L; 95% CI: 0.04 to 0.37). There was no significantly heterogeneity in either of these studies (I^2^ = 0%, *p* = 0.987; Table 3).

## 4. Discussion

The meta-analysis results of this study showed that older age, lower BMI, higher APACHE II, and SOFA scores, lower systolic BP and diastolic BP, lower serum creatinine level, and higher serum sodium level increased the risk of in-hospital mortality among critically ill patients who required CRRT. Unsurprisingly, our review showed that older patients were more at risk of in-hospital mortality, which was in line with previous reviews [5,15]. Older adults tend to be vulnerable to AKI, as they often have complex and multiple comorbidities, polypharmacy, and age-related structural and functional changes in their kidneys [26]. Moreover, because of these physiological changes, older patients with AKI may be at greater risk of hemodynamic instability and are more likely to undergo CRRT [27]. Therefore, healthcare professionals should pay attention to older patients after CRRT and take steps to prevent the incidence of death.

Our results also show that BMI was associated with in-hospital mortality. Low BMI levels can indicate malnutrition, which increases the risk of infection and disease. Specifically, protein and energy malnutrition and deficiencies of specific micronutrients (including iron, zinc, and vitamins) increase susceptibility to infection [28]. Malnutrition among in-patients has been associated with adverse clinical outcomes, including increased mortality, re-admissions, and increased length of hospital stay [29]. A recent study found that lower BMI was an independent risk factor for mortality among older AKI patients who received CRRT [30]. However, another previous study reported that obese patients had a higher risk of mortality than non-obese patients [31]. Yet other studies posit that BMI does not affect mortality at all [32,33]. Therefore, further research is needed to determine the relationship between low BMI and in-hospital mortality in critically ill patients who require CRRT.

The APACHE II and SOFA scores are scoring systems that are commonly used in ICUs [34]. Our findings showed that higher APACHE II and SOFA scores were both associated with high-risk of in-hospital mortality among critically ill patients receiving CRRT. The APACHE II is widely used to quantify the severity of illness during a 24-h stay in an ICU [34]. This system consists of three components: Twelve physiological variables, the previous state of the patient’s health, and their age [35]. The SOFA score is a simpler system and is usually used to assess the severity of multiple organ failure, including the respiratory, circulation, renal, neurologic, hepatogenic, and coagulation systems [36]. AKI is a common cause of complex multiple organ failure syndromes among ICU patients [37]. A recent study reported that the SOFA score showed a higher accuracy of mortality prediction among critically ill patients with AKI undergoing CRRT than the APACHE II score [38]. In addition, these scoring systems rely mainly on data obtained early in the course of a patient’s illness. Therefore, there is a need to develop an AKI-specific scoring system for better severity grading and mortality prediction for patients who require CRRT.

With regard to the hemodynamic factors, our review found that lower systolic and diastolic BP predicted in-hospital mortality following CRRT. Our finding was in line with existing evidence that the chief complication of CRRT is hypotension, which can cause major adverse events, such as myocardial infarction and stroke [39,40]. An existing study involving 1743 patients showed that the incidence of hypotension within one hour of starting CRRT was 64.6%, and that the in-hospital mortality rate was as high as 51% [41]. Although CRRT has good hemodynamic tolerance for critically ill patients, reducing patients’ BP and further worsening of their renal function may sometimes be unavoidable during CRRT [42]. Accordingly, close monitoring of hemodynamics is necessary to ensure timely adjustment in response to hemodynamic instability during CRRT. Our study also identified higher serum sodium levels at the initiation of CRRT as a risk factor for increasing in-hospital mortality. This finding is similar to previous work that showed hypernatremia (high sodium) to lead to longer lengths of stay in hospital and a higher risk of mortality, compared with critically ill patients with normonatremia [43]. However, a recent study reported that serum sodium levels 24 to 72 h after CRRT did not predict mortality [44]. The severity of AKI depends on the increase in serum creatinine level, which is one of the important determinants of deciding to initiate renal replacement therapies worldwide [45,46,47]. In contrast, low levels of serum creatinine at CRRT initiation increased the risk of in-hospital mortality, according to this review. This implies that biochemical laboratory data—including serum sodium and creatinine level—should be further investigated to identify their influence on in-hospital mortality in patients requiring CRRT.

Based on the findings of this review, the in-hospital mortality rate after CRRT for critically ill patients ranged from 38.6% to 62.4%. With the exception of one small-sized study of 70 participants [21], most studies included in this review involved about 200 critically ill patients and had high mortality rates of more than 50%. A recent meta-analysis reported that AKI patients who received CRRT had a 21% higher in-ICU mortality than patients with intermittent hemodialysis [48]. Despite this, very few studies have examined the relationship between CRRT and in-hospital mortality. In this review, we identified several factors that are associated with in-hospital mortality in critically ill patients undergoing CRRT, which may be useful in predicting both short- and long-term outcomes. Further, our findings could contribute to developing a standardized assessment tool for determining the prognosis of critically ill patients after CRRT initiation.

There are several potential limitations to our study. First, our findings are limited in their generalizability, due to the small number of participants included and the small number of studies. Second, we only included published literature and peer-reviewed articles written in English. This review may have overlooked related unpublished research or articles written in other languages. Third, there was a difference in the time of measurement of in-hospital mortality rate between the included studies (7, 28, or 90 days). However, it was not possible to determine the difference in timing of measurement and the relationship with in-hospital mortality rate. Finally, as the included studies did not report on psychological factors, such as depression or anxiety, we were unable to detect an association with in-hospital mortality. Therefore, further studies considering the effect of psychological factors on in-hospital mortality for critically ill patients receiving CRRT are needed.

## 5. Conclusions

This study systematically reviewed multiple modifiable predictors that are independently related to a higher risk of in-hospital mortality among critically ill patients undergoing CRRT. These predictors can contribute to developing a standardized assessment tool for the prognosis of critically ill patients after CRRT initiation. Future large-scale cohort studies are required to confirm our results.

## Figures and Tables

**Table 1:** Characteristics of studies included (*N* = 6).

Authors (Year)/Country or Territories	Study Design	Follow-Up Period (Months)	Sample Characteristics		Indication for Initiation of CRRT	CRRT Modality	In-Hospital Mortality Rate (%)	NOS Quality
			Survivors	Non-Survivors				
Lin et al. (2009)/Taiwan [20]	Prospective	60	*n* = 137 Mean age: 61.1 ± 14.8 (years) Male: 85 (62.0%) Female: 52 (38.0%)	*n* = 205 Mean age: 65.9 ± 15.5 (years) Male: 119 (58.0%) Female: 86 (42.0%)	Azotemia (BUN 80 mg/dL and serum creatinine 2 mg/dL, without evidence of dehydration), uremic symptoms, fluid overload refractory to diuretic use with a central venous pressure 14 mm Hg or pulmonary edema with a PaO_2_/FiO_2_ 300 mmHg, hyperkalemia (serum K 5.5 mmol/L) refractory to medical treatment, oliguria(urine amount 200 mL/8 h) refractory to diuretics, metabolic acidosis (pH 7.2 in arterial blood gas)	CVVH	59.9 (90 days)	9
Kritmetapak et al. (2016)/Thailand [21]	Prospective	13	*N*: 27 Mean age: 57.3 ±16.8 (years) Male: 20 (74.1%) Female: 7 (25.9%)	*N*: 43 Mean age: 62.8 ± 16.8 (years) Male: 27 (62.8%) Female: 16 (37.2%)	Hemodynamically unstable patients with refractory fluid overload, severe hyperkalemia, severe metabolic acidosis, severe azotemia, and uremic symptoms	CVVH	38.6 (28 days)	8
Lu et al. (2016)/China [22]	Retrospective	13	*N*: unreported Mean age: 57 ± 14.4 (years) Male: unreported Female: unreported	*N*: unreported Mean age: 57 ± 14.4 (years) Male: unreported Female: unreported	Eliminating inflammatory mediators, cytokines, alleviating edema, protecting renal function	CVVH, CVVHDF	Unreported (28 days)	8
Cho et al. (2018)/Korea [23]	Retrospective	60	*N* = 128 Mean age: 64 ± 14 (years) Male: 79 (61.7%) Female: 49 (38.35)	*N* = 212 Mean age: 69 ± 12 (years) Male: 135 (63.7%) Female: 77 (36.3%)	Oliguria (urine output < 100 mL in a six-hour period and unresponsive to fluid resuscitation), serum potassium concentration > 6.5 mmol/L, severe acidemia (pH < 7.2), or presence of severe organ edema (e.g., pulmonary edema), severe sepsis associated with acute organ dysfunction and septic shock as sepsis with acute circulatory failure characterized by persistent arterial hypotension	CVVHDF	62.4 (28 days)	7
Kee et al. (2018)/Korea [24]	Retrospective	17	*N* = 110 Mean age: 65.7 ± 15.3 (years) Male: 72 (65.5%) Female: 38 (34.5%)	*N* = 130 Mean age: 65.9 ± 14.2 (years) Male: 78 (60%) Female: 52 (40%)	Medically intractable or persistent electrolyte imbalance and/or metabolic acidosis, and decreased urine output with volume overload and/or progressive azotemia	CVVHDF	54.2 (7 days)	8
Keleshian et al. (2020)/USA [25]	Retrospective	109	*N* = 93 Mean age: 61.5 (years) Male: 63 (67.7%) Female: 30 (32.3%)	*N* = 105 Mean age: 64.8 (years) Male: 66 (62.9%) Female: 39 (37.1%)	Unreported	Unreported	53.0 (in-hospital)	8

Note. CRRT = continuous renal replacement therapy; NOS = Newcastle-Ottawa scale; BUN = blood urea nitrogen; CVVH = continuous venovenous hemofiltration; CVVHDF = continuous venovenous hemodiafiltration.

**Table 2:** Risk factors for hospital mortality among critically ill patients who received CRRT.

Risk Factors	No. of Studies	No. of Participants	OR/SMD	95% CI	I^2^ (%)	*p*-Value
Demographic characteristics						
Age (years)	4	992	0.26 *	0.07 to 0.44	47.3	0.127
BMI (kg/m^2^)	3	652	−0.17 *	−0.33 to −0.01	3.7	0.354
Male	5	1190	0.87	0.69 to 1.11	0	0.080
Female	5	1190	1.15	0.90 to 1.46	0	0.080
Severity scoring						
APACHE II	3	752	1.05 *	0.36 to 1.75	94.0	<0.001
SOFA	2	752	1.06 *	0.61 to 1.51	85.3	<0.001
Comorbidities						
Diabetes mellitus, yes	4	848	0.79	0.59 to 1.07	0	0.463
Hypertension, yes	3	650	1.11	0.67 to 1.86	53.0	0.119
Heart failure, yes	3	778	1.09	0.76 to 1.57	0	0.668
Liver disease, yes	2	410	1.94	0.77 to 4.90	50.1	0.157
Sepsis, yes	4	992	1.55	0.86 to 2.77	60.0	0.058
CAD, yes	2	268	0.61	0.33 to 1.10	0	0.879
COPD, yes	2	438	0.96	0.41 to 2.24	0	0.892
Hemodynamic and clinical characteristics						
Systolic BP (mmHg)	2	580	−0.38 *	−0.55 to −0.22	1.2	0.314
Diastolic BP (mmHg)	2	580	−0.77 *	−0.43 to −0.10	0	0.555
Hemoglobin (g/dL)	2	580	0.04 *	−0.12 to 0.21	0	0.975
White blood cell (10^3^/mL)	2	580	0.06 *	−0.16 to 0.27	40.2	0.196
Platelet (10^3^/mL)	2	580	−0.25 *	−0.72 to 0.23	87.6	0.005
Serum creatinine (mg/dL)	3	752	−0.34 *	−0.48 to −0.19	0	0.541
Serum sodium (mmol/L)	2	580	0.21 *	0.04 to 0.37	0	0.987
Serum potassium (mmol/L)	2	580	−0.08 *	−0.24 to 0.09	0	0.427
Serum calcium (mg/dL)	2	580	0.08 *	−0.21 to 0.36	65.1	0.090
Serum phosphate (mg/dL)	2	580	0.14 *	−0.22 to 0.50	78.1	0.033
Total bilirubin (mg/dL)	2	580	0.21 *	−0.07 to 0.49	63.8	0.096
Reasons for CRRT						
Fluid overload	3	610	1.22	0.86 to 1.74	0	0.488
Severe acidosis	3	752	1.20	0.58 to 2.48	27.4	0.252
Oliguria	3	752	0.83	0.47 to 1.47	47.4	0.149
ICU mechanical assist device						
ECMO or IABP, yes	2	540	1.45 *	0.77 to 2.88	61.5	0.107

Note. * SMD = standardized mean difference; OR = odds ratio; CI = confidence interval; BMI = body mass index; APACHE II = acute physiology and chronic health evaluation; SOFA = the sequential organ failure evaluation; CAD = coronary artery disease; COPD = chronic obstructive pulmonary disease; BP = blood pressure; CRRT = continuous renal replacement therapy; ECMO = extracorporeal membrane oxygenation; IABP = intra-aortic balloon pump.

**Table 3:** Forest plot of risk factors.

**Age (Years)**						
**Study**	**SMD**	**Lower Limit**	**Upper Limit**	**Z-Value**	***p*-Value**	**SMD and 95% CI**
Lin et al. (2009) [20]	0.32	0.10	0.53	2.84	0.005	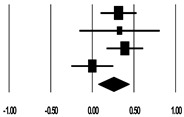
Kritmetapak et al. (2016) [21]	0.33	−0.16	0.81	1.33	0.185	
Cho et al. (2018) [23]	0.39	0.17	0.61	3.46	0.001	
Kee et al. (2018) [24]	0.00	−0.25	0.25	0.00	1.000	
Total	0.26	0.07	0.44	2.71	0.007	
I^2^ = 47.3%, *p* = 0.127						
**BMI (kg/m^2^)**						
**Study**	**SMD**	**Lower Limit**	**Upper Limit**	**Z-Value**	***p*-Value**	**SMD and 95% CI**
Lin et al. (2009) [20]	−0.26	−0.48	−0.05	−2.39	0.017	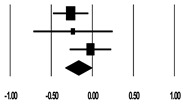
Kritmetapak et al. (2016) [21]	−0.24	−0.72	0.25	−0.96	0.338	
Kee et al. (2018) [24]	−0.02	−0.28	0.23	−0.18	0.857	
Total	−0.17	−0.33	−0.01	−2.07	0.038	
I^2^ = 3.7%, *p* = 0.354						
**APACHE II Score**						
**Study**	**SMD**	**Lower Limit**	**Upper Limit**	**Z-Value**	***p*-Value**	**SMD and 95% CI**
Lin et al. (2009) [20]	0.64	0.42	0.86	5.64	<0.001	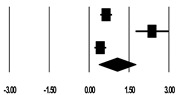
Kritmetapak et al. (2016) [21]	2.36	1.74	2.98	7.47	<0.001	
Cho et al. (2018) [23]	0.42	0.20	0.64	3.70	<0.001	
Total	1.05	0.36	1.75	2.96	0.003	
I^2^ = 94.0%, *p* < 0.001						
**SOFA Score**						
**Study**	**SMD**	**Lower Limit**	**Upper Limit**	**Z-Value**	***p*-Value**	**SMD and 95% CI**
Lin et al. (2009) [20]	0.83	0.60	1.05	7.19	<0.001	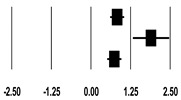
Kritmetapak et al. (2016) [21]	1.87	1.32	2.47	6.47	<0.001	
Cho et al. (2018) [23]	0.74	0.51	0.97	6.40	<0.001	
Total	1.06	0.61	1.51	4.60	<0.001	
I^2^ = 85.3%, *p* = 0.001						
**Systolic BP (mmHg)**						
**Study**	**SMD**	**Lower Limit**	**Upper Limit**	**Z-Value**	***p*-Value**	**SMD and 95% CI**
Cho et al. (2018) [23]	−0.31	−0.53	−0.09	−2.75	0.006	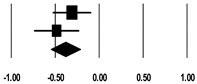
Kee et al. (2018) [24]	−0.48	−0.74	−0.23	−3.68	<0.001	
Total	−0.38	−0.55	−0.22	−4.46	<0.001	
I^2^ = 1.2%, *p* = 0.314						
**Diastolic BP (mmHg)**						
**Study**	**SMD**	**Lower Limit**	**Upper Limit**	**Z-Value**	***p*-Value**	**SMD and 95% CI**
Cho et al. (2018) [23]	−0.22	−0.44	−0.01	−2.00	0.046	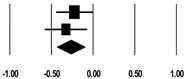
Kee et al. (2018) [24]	−0.33	−0.58	−0.01	−2.50	0.013	
Total	−0.27	−0.43	−0.10	−3.14	0.002	
I^2^ = 0%, *p* = 0.555						
**Serum Creatinine (mg/dL)**						
**Study**	**SMD**	**Lower Limit**	**Upper Limit**	**Z-Value**	***p*-Value**	**SMD and 95% CI**
Lin et al. (2009) [20]	−0.29	−0.51	−0.07	−2.61	0.009	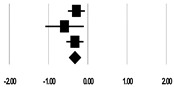
Kritmetapak et al. (2016) [21]	−0.59	−1.08	−0.10	−2.37	0.018	
Cho et al. (2018) [23]	−0.33	−0.55	−0.11	−2.92	0.004	
Total	−0.34	−0.48	−0.19	−4.44	<0.001	
I^2^ = 0%, *p* = 0.541						
**Serum Sodium (mmol/L)**						
**Study**	**SMD**	**Lower Limit**	**Upper Limit**	**Z-Value**	***p*-Value**	**SMD and 95% CI**
Cho et al. (2018) [23]	0.20	−0.02	0.42	1.82	0.069	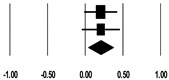
Kee et al. (2018) [24]	0.21	−0.05	0.46	1.59	0.111	
Total	0.21	0.04	0.37	2.42	0.016	
I^2^ = 0%, *p* = 0.987						

Note. SMD = standardized mean difference; CI = confidence intervals; BMI = body mass index; APACHE II = acute physiology and chronic health evaluation; SOFA = the sequential organ failure evaluation; BP = blood pressure.

## Supplementary Materials

The following are available online at https://www.mdpi.com/1660-4601/17/23/8781/s1. Table S1. Search Results.
