# Supplementary material for: Factors Associated with In-Hospital Mortality after Continuous Renal Replacement Therapy for Critically Ill Patients: A Systematic Review and Meta-Analysis
Source: Int J Environ Res Public Health. 2020 Nov 26;17(23):8781. doi: 10.3390/ijerph17238781 (PMC7730748; doi:10.3390/ijerph17238781)
Supplement: Supplementary file 1 [file ijerph-17-08781-s001.pdf]

Table 1. Search Results.

Database	Number	Search query	Results
Pubmed	#1	("Renal Insufficiency"[Mesh] OR Renal Insufficienc*[ti] OR Renal Failure*[ti] OR Kidney Failure*[ti] OR Kidney insufficienc*[ti] OR "Acute Kidney Injury"[Mesh] OR "Acute Kidney injury"[ti])	176,100
	#2	("Continuous Renal Replacement Therapy"[Mesh] OR "Continuous Renal Replacement Therapy"[ti] OR CRRT[ti] OR "Continuous hemofiltration"[ti] OR "Continuous RRT"[ti] OR RRT[ti])	1,575
	#3	("Intensive Care Units"[Mesh] OR Intensive Care Unit*[ti] OR Critical Care Unit*[ti])	89,718
	#4	#1 AND #2 AND #3	144
	#5	#4 AND (("0001/01/01"[PDAT] : "2020/06/30"[PDAT]) AND "humans"[MeSH Terms] AND English[lang])	**134**
EMBASE	#1	kidney failure'/de OR 'kidney failure':ti OR 'kidney insufficiency':ti OR 'maternal kidney failure':ti OR 'renal failure':ti OR 'renal insufficiency':ti OR 'terminal kidney failure':ti OR 'acute kidney failure'/de OR 'acute kidney failure':ti OR 'acute kidney injury':ti OR 'acute kidney insufficiency':ti OR 'acute renal failure':ti OR 'acute renal insufficiency':ti OR 'kidney acute failure':ti OR 'kidney failure, acute':ti OR 'kidney insufficiency, acute':ti OR 'renal insufficiency, acute':ti	238,479
	#2	continuous renal replacement therapy'/de OR 'crrt':ti OR 'continuous renal replacement therapy':ti OR 'continuous hemofiltration'/de OR 'cvvh':ti OR 'continuous arterio-venous haemofiltration':ti OR 'continuous arterio-venous hemofiltration':ti OR 'continuous arteriovenous haemofiltration':ti OR 'continuous arteriovenous hemofiltration':ti OR 'continuous haemofiltration':ti OR 'continuous hemofiltration':ti OR 'continuous veno-venous haemofiltration':ti OR 'continuous veno-venous hemofiltration':ti OR 'continuous venovenous haemofiltration':ti OR 'continuous venovenous hemofiltration':ti OR 'continuous rrt':ti OR rrt:ti	8,132
	#3	intensive care unit'/de OR 'gicu':ti OR 'gicus':ti OR 'icu`s':ti OR 'close attention unit':ti OR 'combined medical and surgical icu':ti OR 'combined surgical and medical icu':ti OR 'critical care unit':ti OR 'general icu':ti OR 'intensive care department':ti OR 'intensive care unit':ti OR 'intensive care units':ti OR 'intensive therapy unit':ti OR 'intensive treatment unit':ti OR 'medical-surgery icu':ti OR 'medical/surgical icu':ti OR 'medical/surgical icus':ti OR 'medico-surgical icu':ti OR 'mixed medical and surgical icu':ti OR 'mixed surgical and medical icu':ti OR 'respiratory care unit':ti OR 'respiratory care units':ti OR 'special care unit':ti OR 'surgery/medical icu':ti OR 'surgical-medical icus':ti OR 'surgical/medical icu':ti OR 'unit, intensive care':ti	164,684
	#4	#1 AND #2 AND #3	919
	#5	#4 AND ([article]/lim OR [article in press]/lim OR [conference paper]/lim OR [conference review]/lim OR [review]/lim) AND [english]/lim AND [humans]/lim	**576**
CINAHL	#1	(MM "Renal Insufficiency+")	32,279
	#2	(MM "Kidney Failure, Acute+")	7,630
	#3	TI Renal Insufficienc* OR Renal Failure* OR Kidney Failure* OR Kidney insufficienc* OR "Acute Kidney injury"	9,026
	#4	#1 OR #2 OR #3	34,325
	#5	(MM "Continuous Renal Replacement Therapy+")	917
	#6	TI "Continuous Renal Replacement Therapy" OR CRRT OR "Continuous hemofiltration" OR "Continuous RRT" OR RRT	845
	#7	#5 OR #6	1,540
	#8	(MM "Intensive Care Units+")	25,833
	#9	TI Intensive Care Unit* OR Critical Care Unit*	18,501
	#10	#8 OR #9	34,895
	#11	#4 AND #7 AND #10	32
	#12	#12 AND human	**18**
Cochrane	#1	MeSH descriptor: [Renal Insufficiency] explode all trees	8,758
	#2	MeSH descriptor: [Acute Kidney Injury] explode all trees	1,400
	#3	(Renal Insufficienc* OR Renal Failure* OR Kidney Failure* OR Kidney insufficienc* OR "Acute Kidney injury"):ti	3,835
	#4	#1 OR #2 OR #3	11,039
	#5	MeSH descriptor: [Continuous Renal Replacement Therapy] explode all trees	5
	#6	(CRRT OR "Continuous Renal Replacement Therapy" OR "Continuous hemofiltration" OR "Continuous RRT" OR RRT):ti	239
	#7	#5 OR #6	242
	#8	MeSH descriptor: [Intensive Care Units] explode all trees	3,477

	#9	(Intensive Care Unit* OR Critical Care Unit*):ti	2,300
	#10	#8 OR #9	5,212
	#11	#4 AND #7 AND #10	**9**
**Web of Science**	#1	(("Renal Insufficienc*" OR "Renal Failure*" OR "Kidney Failure*" OR "Kidney insufficienc*" OR "Acute Kidney injury") AND ("Continuous Renal Replacement Therapy" OR CRRT OR "Continuous hemofiltration" OR "Continuous RRT" OR RRT) AND ("Intensive Care Unit*" OR "Critical Care Unit*"))	**15**
